# The Utility of Immuno-Nutritional Scores in Patients with Testicular Germ Cell Tumors

**DOI:** 10.3390/diagnostics14192196

**Published:** 2024-10-01

**Authors:** Uros Bumbasirevic, Milos Petrovic, Vesna Coric, Nikola Lisicic, David Obucina, Milica Zekovic, Bogomir Milojevic, Nenad Vasilic, Vladimir Vasic, Marko Zivkovic, Nebojsa Bojanic, Aleksandar Janicic

**Affiliations:** 1 Clinic of Urology, University Clinical Center of Serbia, 11000 Belgrade, Serbia; milospet93@gmail.com (M.P.); nikola.lisicic1@hotmail.com (N.L.); davidobucina@gmail.com (D.O.); em2bogomir@yahoo.com (B.M.); drnenadvasilic@gmail.com (N.V.); markoziv91@gmail.com (M.Z.); bojanicnebojsa@gmail.com (N.B.); 2 Faculty of Medicine, University of Belgrade, 11000 Belgrade, Serbia; 3 Institute of Medical and Clinical Biochemistry, Faculty of Medicine, University of Belgrade, 11000 Belgrade, Serbia; drcoricvesna@gmail.com; 4 Center of Excellence for Redox Medicine, Faculty of Medicine, University of Belgrade, 11000 Belgrade, Serbia; 5 Centre of Research Excellence in Nutrition and Metabolism, Institute for Medical Research, National Institute of Republic of Serbia, University of Belgrade, 11000 Belgrade, Serbia; zekovicmilica@gmail.com; 6 Department of Urology, University Medical Center Zvezdara, 11000 Belgrade, Serbia; vladimirdrvasic@gmail.com

**Keywords:** immuno-nutritional scores, HALP, PNI, testicular GCT

## Abstract

Background: Hemoglobin, Albumin, Lymphocyte, and Platelet Score (HALP) is an accessible score that is easily reproducible from routine laboratory testing while also reflecting patients’ immune-nutritional status. Along with other immuno-nutritional scores, such as the Prognostic Nutrition Index (PNI), HALP has been associated with a number of clinical and pathological features. The goal of our study was to evaluate the prognostic utility of HALP and PNI scores in testicular germ cell cancer (GCT) patients. Methods: This case-only study included 203 testicular GCT patients who were classified according to the disease stage and HALP and PNI cut-offs. Complete blood count and albumin concentration were routinely determined. Results: The values of HALP and PNI significantly differed among different clinical stages (*p* < 0.05). Moreover, they clearly exposed a significantly higher risk of advanced clinical stage development for those testicular GCT patients with lower values of HALP and PNI (*p* < 0.05). Finally, lower score levels were associated with larger tumor size (*p* < 0.05). Conclusion: Our investigation could provide evidence that specific immune-nutritional scores can help distinguish individuals diagnosed with testicular GCT who are more likely to be identified with advanced disease stages.

## 1. Introduction

Testicular cancer (TC) is a relatively uncommon malignancy, although it represents the most common malignant tumor in young men between the ages of 20 and 40 years. Despite a steady increase in incidence rates over the previous three decades, testicular cancer presents one of the most curable genitourinary malignancies due to the high efficacy of the multidisciplinary treatment approach that incorporates surgery, cisplatin-based chemotherapy, and radiation [1]. Nevertheless, TC still remains an important contributor to cancer-related mortality among men in this age group worldwide, particularly in countries with lower socioeconomic levels [2]. Approximately 95% of testicular cancers are classified as germ cell tumors (GCT), with the remaining 5% mostly consisting of sex cord tumors. Seminomas account for 55–60% of GCT, whereas the remaining 40–45% are comprised of non-seminomatous germ cell tumors (NSGCT) [3].

An increasing amount of data suggests a complex interrelationship between chronic inflammation, nutritional status, and the development and progression of cancer [4,5]. All aspects of carcinogenesis, including the induction of genomic instability and neoplastic transformation, angiogenesis, inhibition of apoptosis, and progression to metastasis, may be correlated with chronic and dysregulated inflammation [6]. The significance of immuno-nutritional status in oncological patients has been increasingly recognized. Immune system dysfunction and nutritional impairment are more common in cancer patients due to their high metabolic requirements and protracted catabolic state [7]. Oncological patients with deficient immuno-nutritional status experience reduced tolerance and responsiveness to chemotherapy, higher occurrence of chemotherapy side effects, and longer hospital stays [8]. Furthermore, those patients have an increased risk of disease progression and inferior oncological outcomes [9]. Over time, several promising prognostic indices of systemic inflammation, such as neutrophile-to-lymphocyte ratio (NLR), platelet-to-lymphocyte ratio (PLR), and systemic immune-inflammation index (SII), have been developed and studied in various cancer types, including TC [10,11].

The Hemoglobin, Albumin, Lymphocyte, Platelet Score (HALP), and the prognostic nutritional index (PNI) are emerging prognostic biomarkers that comprehensively integrate indicators of systemic inflammation and nutritional state and can be readily obtained and accurately determined using routine laboratory measurements [12,13]. Platelets have been recognized as significant facilitators of angiogenesis and metastatic progression, along with protecting cancer cells from immune surveillance [14]. Lymphocytes are key regulators of tumor-suppressive mechanisms of adaptive immunity, including cancer immunosurveillance, senescence surveillance, and cancer immunoediting. Consequently, lymphocyte depletion can be associated with deficient control of tumor growth and disease progression [15]. Anemia, the most prevalent hematological abnormality in cancer patients, is caused by a combination of cancer-related and non-cancer-related mechanisms. It has been recognized as a strong predictor of disease progression and reduced cancer-specific survival (CSS) [16]. Lastly, increased metabolic requirements and the production of multiple proinflammatory cytokines result in the development of cancer-associated hypoalbuminemia, which enhances the risk of cancer cachexia, a well-established prognosticator of poor treatment response and survival [17]. By incorporating previously mentioned variables, these scores correlate with a reduced immuno-nutritional state and could have significant implications in regard to immuno-nutritional function risk stratification and treatment optimization in cancer patients.

The clinical and prognostic significance of immuno-nutritional scores has been increasingly investigated in various cancers. The decreased values of HALP have been associated with poor overall survival (OS) in lung, breast, gastrointestinal, urological (urothelial and prostate cancer) and gynecological cancers [9]. The 2023 meta-analysis, which encompassed 28 studies and a total of 13,110 patients with solid tumors, determined that the HALP score is a negative predictor of OS, CSS, and recurrence-free survival (RFS) [18]. An unfavorable prognosis is linked to a low level of PNI, based on meta-analyses of different tumor types [19,20]. Furthermore, it has predictive value for patients undergoing a variety of cancer therapies, including immunotherapy [21,22].

However, there has been limited research on the significance of immuno-nutritional state and HALP and PNI scores in testicular cancer patients. Therefore, the aim of our study was to determine the prognostic utility of preoperative HALP and PNI scores in TC.

## 2. Materials and Methods

### 2.1. Study Population

During the period between 2020 and 2024, a total of 229 consecutive newly diagnosed testicular GCT patients were treated at the Clinic of Urology, University Clinical Centre of Serbia, Belgrade. The International Union Against Cancer (UICC) standard tumor, node, metastasis (TNM) classification [23] guided clinical staging after CT imaging and tumor marker analysis. Patients that had the presence of other malignant tumors, preoperative evidence of infection, significant inflammatory conditions, immune system disorders, or disorders that could affect albumin levels, incomplete clinical data, or preoperative laboratory parameters were excluded. As a result, the final study group consisted of 203 patients.

### 2.2. Data Collection and Definition

Standard epidemiological characteristics such as age, BMI, smoking status, and comorbidities, as well as pathological characteristics like stage, tumor, primary type, tumor size, tumor multifocality, and presence of lympho-vascular invasion (LVI) were collected. The concept of “multifocality” refers to the presence of a distinct tumor focus composed of a group of malignant cells exceeding 1 mm, which can be distinguished from the primary tumor mass. The Charlson Comorbidity Index (CCI) was determined by a thorough medical evaluation utilizing a standardized scoring procedure [24]. The laboratory parameters were obtained routinely within one day prior to radical orchiectomy, which included counts of lymphocytes and platelets as well as measurements of hemoglobin and albumin levels. The HALP score was calculated as follows, hemoglobin (g/L) × albumin (g/L) × lymphocytes (/L)) / platelets (/L) while PNI (prognostic nutritional index) was calculated as follows: 10 × serum albumin (g/dL) + 5 × lymphocytes (109/L).

### 2.3. Ethical Considerations

The study was conducted in accordance with the institutional ethical board standards (Approval number 717/9, University Clinical Centre of Serbia, Serbia) and the principles of the Declaration of Helsinki.

### 2.4. Statistical Analysis

The statistical data analysis was conducted using IBM SPSS Statistics 22 (SPSS Inc. Chicago, IL, USA) and R software 4.4 (https://www.r-project.org/, accessed on 3 August 2024). The Mann–Whitney U test was used to evaluate the disparities in continuous data that exhibited a non-normal distribution. χ^2^ test was used for categorical variables. ROC curve analysis was conducted to identify the ideal cut-off values for each score using the Youden index. The risk of higher clinical stage development was computed by odds ratios (OR) and 95% confidence intervals (CI) by logistic regression analysis. The statistical hypotheses were analyzed using a significance level of 0.05.

## 3. Results

Table 1 and Table 2 summarize the descriptive data of all recruited individuals with testicular GCT. As indicated in Table 1, the majority of patients were diagnosed with seminoma (59%) at clinical stage I (73%). Almost half of the patients had lympho-vascular invasion (47%).

The median calculated *Charlson Comorbidity Index* (CCI) for the whole cohort, as well as within different clinical stages, was 0-signifying the minimal burden that could contribute to the inflammation process (Table 2). There was no notable disparity in terms of median BMI values between testicular GCT patients with lower tumor stage (Stage I) and higher tumor stage (Stages II + III) (*p* > 0.05). What is more, when the patients were dichotomized according to a BMI value of 30 kg/m^2^, the obtained analysis did not exhibit any significant difference between the two groups (*p* > 0.05). However, the smoking status alone increased the risk of higher-stage development almost two times (OR = 1.957, 95% CI = 1.035–3.698, *p* = 0.039). Similarly, tumor size, as one of the most speculated prognostic factors, seems to raise the stage development risk by 3.9 times (95% CI = 1.973–7.681, *p* < 0.001). On the other hand, the presence of multifocality seems to significantly lower the risk of higher-stage development (OR = 0. 306, 95% CI = 0.102–0.919, *p* = 0.035).

Patients with higher stage were found to have significantly altered values of absolute number of platelets, as well as greatly changed concentrations of hemoglobin and albumin (*p* < 0.05, Table 3). All aforementioned parameters have been integrated into the calculation of HALP and PNI scores, as indicated by the formulas provided below in Table 3. Indeed, Table 3 presents the disparity in preoperative scores between testicular GCT patients with lower tumor stage (Stage I) and higher tumor stages (Stages II + III) (*p* < 0.05, Table 3).

**Figure 1:**
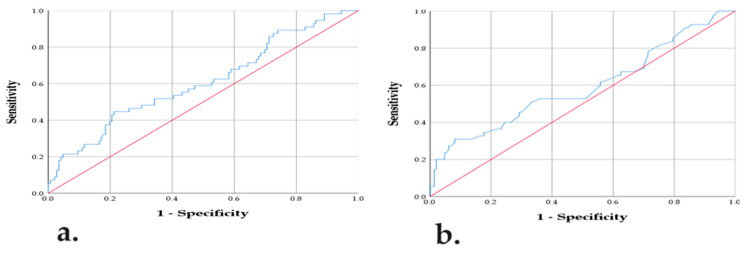
ROC plot for HALP (**a**). ROC plot for PNI (**b**). ROC-receiver operating curve; HALP-hemoglobin, albumin, lymphocytes, and platelets score; PNI-prognostic nutritional index. Blue line: represents the different sensitivity/specificity pair value for the classification threshold. Red line: Diagonal line.

The findings of the ROC analysis are displayed in Table 4 and Figure 1. Assessed inflammatory-nutritional scores exhibited substantial prognostic ability for metastatic illness prior to orchiectomy. Namely, the HALP and PNI cut-offs, along with the respective area under the curve (AUC), sensitivity, and specificity, indicate the discriminating potential of the assessed scores (AUC > 0.5, *p* < 0.05), which is depicted in Figure 1.

The association between various clinicopathological parameters and immuno-nutritional scores is displayed in Table 5. The HALP and PNI cut-off values were employed to categorize patients into low and high-score groups. Within the group of patients with clinically advanced disease (CS II + III), 49% of them exhibited a low HALP score. In contrast, among patients with localized disease (CSI), a low HALP score was found in 20% of patients. This difference was shown to be statistically significant (*p* < 0.0001), indicating that a preoperative low HALP score may be a prognosticator of the development of advanced disease. Similar findings were observed for PNI (*p* < 0.0001). In comparison to clinical stage I disease, lower PNI levels were statistically significantly more frequent in later stages of the disease (29% vs. 8%, *p* = 0.001, and 51% vs. 23%, *p* < 0.0001). Further analysis revealed that low HALP and PNI scores were statistically significantly associated with tumors >4 cm (*p* = 0.004 for HALP, and *p* < 0.0001 for PNI). However, no such association was obtained when the patients were dichotomized according to BMI, LVI, and multifocality status (*p* < 0.05).

Ultimately, we used the dichotomization of the patients with testicular GCT according to the score values to evaluate the risk of higher-stage development using two logistic regression models (Table 6). The first model calculated the crude odds ratio (OR1), whereas the second model was adjusted for characteristics that altered the risk of higher-stage development (namely, tumor size, smoking status, and multifocality), as shown in Table 6. The results of such analysis indicated that the patients with testicular GCT who had lower values of preoperative HALP scores (values < 42.56) were running the risk of higher clinical stage development (OR1 = 3.793 (1.954–7.363), *p* < 0.001) than patients with testicular GCT who had higher values of preoperative HALP score (values > 42.56). This risk was even more pronounced when the adjusted analysis was computed (OR2= 4.161, 95% CI = 1.862–9.269, *p* = 0.001). The increased risk of developing higher clinical stage was also found for patients with low PNI (OR1 = 4.650, 95% CI = 2.030–10.650, *p* < 0.001 and OR2 = 5.556, 95% CI = 1.969–15.677, *p* = 0.001) as opposed to those with higher PNI (values > 52.5).

## 4. Discussion

Up-regulation of numerous inflammatory mediator molecules that leads to cancer progression can be mirrored by the determination of certain routine laboratory parameters that can be further incorporated into integrative scores, such as HALP and PNI. On the other hand, nutrition status has a significant role in the development and progression of cancer and has a direct impact on the survival of patients both during and after receiving final treatments. In this particular study, we assessed the role of immuno-nutritional scores in patients with testicular GCT. Indeed, our results have indicated the altered values of both hematological (lymphocytes, platelets) and biochemical laboratory parameters (hemoglobin and albumin) between disease clinical stages of patients with testicular GCT. These particular laboratory parameters were additionally integrated into immuno-nutritional scores (HALP and PNI).

HALP score has been associated with various clinical and pathological parameters in prior research [9]. One almost consistent finding is a link between HALP and an elevated risk of advanced-stage disease at the diagnosis [12,25,26,27,28,29]. The PNI was initially employed to assess the perioperative nutritional status in gastrointestinal surgery [13]. However, it has progressively evolved into a prognostic indicator for predicting outcomes in many types of cancer [30]. Similarly to HALP, multiple studies have found that lower preoperative levels of PNI are associated with higher stages of malignancy [31,32]. Cancer-induced anemia has a complex and multifactorial pathogenesis. It can occur due to cancer-related blood loss, bone marrow tumor infiltration, or radiotherapy- and chemotherapy-induced erythropoiesis inhibition. In other cases, the etiology of cancer-induced anemia cannot be discerned, and it is referred to as anemia of chronic disease [16]. The primary underlying mechanism of this form of anemia has been proposed to be the increased synthesis of hepcidin during inflammation and subsequent dysregulation of iron homeostasis, which is predominantly mediated by interleukin-6 [33]. Hence, anemia can be a common finding among cancer patients with an advanced stage of the disease [34]. Preoperative anemia was detected in 13% of our patients, being significantly more prevalent in patients with clinically advanced disease (25% vs. 9%, *p* = 0.002). Likewise, as part of the systemic inflammatory response, interleukin-6 effects result in decreased albumin production [35]. Moreover, increased catabolism and chronic consumption state are important features of cancer progression, all leading to the development of hypoalbuminemia [7]. Important aspects of metastatic development include evading intrinsic growth control and senescence surveillance by tumor cells and altering cancer immunoediting to low immunogenicity. These characteristics are especially prominent in the state of lymphopenia [15]. Extensive lymphoid infiltrates with a high proportion of activated cytotoxic lymphocytes in testicular seminoma have been postulated as one of the primary processes contributing to a better prognosis in this tumor [36]. As a result, it is reasonable to presume that lymphopenia in these patients can be related to an advanced stage of disease. Platelets, the last component of the HALP score, may also contribute to cancer progression, principally by protecting tumor cells in the circulation and enhancing tumor angiogenesis and invasion [14]. By encompassing these hematological and nutritive variables, the HALP and PNI scores appear to have the potential to be a robust prognosticator of advanced disease in cancer patients. Although not examined in this context testicular cancer patients, the HALP score has been studied in other malignancies for its prognostic value. For instance, Chen et al. conducted a retrospective cohort study in 2015 to assess the predictive value of the preoperative HALP score in 1332 gastric cancer patients. Univariate analysis found that patients with high HALP values were substantially more prevalent in the T1-T1, NO, and MO stages than individuals with low HALP values. Furthermore, in multivariate analysis, the T stage was independently associated with the preoperative HALP score [12]. Similarly, the low HALP score was related to the high TNM stage and lymph node metastases in pancreatic cancer [25]. The prognostic significance of pre-treatment HALP score in relation to clinically advanced disease was also identified in gynecological malignancies [26,27]. In a prospective cohort study of 439 patients with endometrial cancer, low values of HALP score were significantly more prevalent in late FIGO stages [26]. Similarly, Leetanaporn and colleagues reported that among patients with cervical cancer, a low HALP score was linked with a higher stage of disease [27]. Among urological malignancies, only two studies have examined the relationship between HALP and the stage of the disease [28,29]. Patients with renal cell carcinoma (RCC) who had a decreased HALP level showed a correlation with multiple adverse histopathological variables, including advanced T stage, presence of lymph node metastases, and distant metastases [28]. Gao et al. conducted a retrospective, multicenter study, including 533 patients with upper tract urothelial carcinoma (UTUC) who had radical nephroureterectomy. The study found that low HALP levels were strongly linked to more advanced pathologic T and N stages [29].

Our study was the first to assess the prognostic relevance of advanced disease presence of the HALP score and PNI in testicular cancer patients. Upon diagnosis, the majority of patients in our study had localized disease (73%), whereas the remaining 27% of patients presented with clinically advanced disease (clinical stage II and III) having statistically lower median levels of HALP score. After determining the HALP cut-off value using ROC analysis (42.56), it was shown that half of the patients in the advanced stage group had lower HALP scores. Ultimately, low levers of HALP were associated with a more than threefold higher risk of presenting with clinically advanced disease compared to patients with high HALP levels. Adjusted analysis revealed that those with low HALP scores, who were also smokers and had tumor size over 4 cm, had a fourfold higher probability of presenting with advanced disease. Although the difference in median PNI values was not as pronounced between the various clinical stages of the diseases, the values did statistically differ with the cut-off value of 52.5 on ROC analysis, potentially distinguishing between the stages. Upon patient dichotomization, similar to the HAPL score, the risk of having clinically advanced disease was higher in patients with low PNI compared to patients with high PNI values, which was later validated in the adjusted model. Indeed, one-third of individuals with Stages II + III had lower levels compared to only 8% of those with Stage I. A similar distribution of low/high PNI score values was shown to be significantly associated with tumor size but not with the presence of LVI or multifocality. Our results were as expected, with tumor size alone (>4 cm) increasing the chance of acquiring advanced clinical stage and lower HALP and PNI being associated with higher tumor size. These findings align with other studies conducted on different forms of cancer, further reinforcing the value of this biomarker as a reliable indicator of cancer progression and late-stage disease. In general, the size of a tumor can be seen as an indicator of its potential aggressiveness and provide significant prognostic information. Regarding testicular cancer, it has been observed that tumor size larger than 4 cm is an important prognostic factor of relapse in patients with stage I seminoma who underwent active surveillance [37]. In addition to invasion of the rete testis, the size of the tumor >4 cm is utilized to classify patients with stage I seminoma into low- and high-risk groups. This categorization is an important tool in deciding if adjuvant therapy is necessary [38]. Multiple studies have examined the potential link between HALP and tumor size in various types of cancer, with contradictory findings. In a landmark study from 2015., Chen and colleagues observed that gastric cancer patients with low HALP scores had statistically significantly larger tumors compared to patients with high HALP scores (5.2 ± 2.6 cm vs. 4.11 ± 2.7 cm, *p* < 0.0001) [12]. Comparable results have been noted in UTUC patients, where large tumors (>3 cm) were significantly more common in patients with low HALP scores [29]. However, these findings have not been confirmed among patients with cervical cancer [27]. Our study aimed to assess the potential correlation between tumor size (>4 cm versus <4 cm) and HALP score in both seminomatous and non-seminomatous GCT patients. Among the subset of patients with low HALP scores, 40% of them had tumors larger than 4 cm, while 21% had tumor size <4 cm, indicating a statistically significant difference (*p* = 0.004). This finding further supports the connection between low HALP values and increased aggressiveness of the tumor.

Historically, BMI has served as a clinical and epidemiological measure of obesity. Despite the acknowledged limitations of BMI, such as its inability to account for the impact of fat-free mass and age on weight, BMI remains an effective tool for evaluating the risk of hypertension, hypercholesterolemia, diabetes, and cancer [39]. Given that a lower BMI may suggest an impaired nutritional state, several authors have examined the correlation between this measure and HALP. Gao et al. found a statistically significant link between a low HALP score and a BMI < 25 kg/m^2^ in patients with UTUC [29]. However, this observation has not been demonstrated in gynecological malignancies [26,27]. As testicular cancer is the most prevalent malignant tumor in men between the ages of 20 and 40, patients in our cohort were young, with a median age of 33 years and a median BMI of 25.4 kg/m^2.^ Our results have shown that 12% of patients had obesity, which was defined as having a BMI > 30 kg/m^2^. In contrast, only 4.6% of patients were seen to have a BMI < 20 kg/m^2^. Given the aforementioned findings, we found no association between PNI and HALP levels and BMI. According to previous research and our own study, the utility of BMI as a tool for evaluating impaired nutritional status in cancer patients appears to be restricted. Other measures, such as muscle index, muscle attenuation, and weight loss, seem to have a superior prognostic ability [40].

In our further analysis, we aimed to assess the possible relationship between preoperative HALP score and PNI and mono- and multifocality of testicular cancer. The preoperative detection of multifocality is of critical importance for patients with testicular cancer who are eligible for testis-sparing surgery in patients with bilateral or single testis tumors since it can modify the treatment approach and lead to radical orchiectomy [41]. Previous research has demonstrated a strong connection between the presence of multifocality in patients with testicular cancer and smaller tumor sizes, with the highest occurrence observed in tumors measuring <2 cm. Moreover, patients with monofocal tumors had a significantly higher rate of tumors larger than 4 cm [42]. However, the presence of multifocality or monofocality did not show a statistically significant link with the HALP and PNI in our study, which limits the use of these scores in this particular clinical context.

Because our group of patients had a low mortality rate, we did not investigate the prediction capabilities of HALP and PNI in relation to survival outcomes in our study. So far, there has been only one study that examined the predictive value of PNI for GCT outcomes [43]. Among their group of 66 patients with metastatic disease, it has been demonstrated that a PNI cut-off of 32 is an independent prognostic indicator for both OS and progression-free survival (PFS).

It is necessary to acknowledge our study’s limitations. Due to the retrospective, single-center methodology of the present study, there can be significant bias in the selection of the data. Furthermore, unlike most prior studies on other malignancies in the current literature, we did not assess the prognostic ability of HALP and PNI in testicular cancer patients with respect to survival outcomes OS and CSS. Nevertheless, it must be recognized that the OS and CSS rates among patients with testicular cancer are remarkably high. This suggests that in order to adequately evaluate the prognostic significance of HALP and PNI, a very large number of patients and long-term follow-ups are required. Another important limitation is the absence of a defined, consensus-based threshold value for HALP, resulting in substantial variation in methodology and data interpretation across different studies. Finally, the disputable effect of both advanced age and gender on HALP values has been avoided, as the study was conducted on young males. Despite the aforementioned limitations, our study brings significant value in the field of immuno-nutritional scores research, being the first study to assess the prognostic significance of these scores in testicular cancer patients and providing multiple valuable findings which confirmation is required in future, prospective, multicenter investigations.

## 5. Conclusions

The interplay between inflammation and cancer progression is well known. Our investigation could provide evidence that specific immune-nutritional scores can help distinguish individuals diagnosed with testicular GCT who are more likely to be identified with advanced disease stages. In this particular study, the values of HALP and PNI clearly indicated the discriminating potential in terms of patients’ staging and exposed a higher risk of advanced clinical stage development for those testicular GCT patients with lower values of HALP and PNI. Finally, lower score levels were associated with larger tumor size. Still, additional extensive investigations with extended follow-up periods are necessary to confirm the intricate connections between systemic inflammation and nutritional status in this group of patients.

## Figures and Tables

**Table 1:** Clinicopathological characteristics of patients with Testicular GCT.

Parameters ^1^	Patients with Testicular GCT, *n* = 203
Tumor type, *n* (%)	
Seminoma	119 (59)
Non-seminoma	84 (41)
Clinical stage, *n* (%)	
I	148 (73)
II	38 (19)
III	17 (8)
Lympho-vascular invasion, *n* (%)	
No	93 (46)
Yes	96 (47)

^1^ Available data; GCT-germinative cell tumor.

**Table 2:** Epidemiological and pathological characteristics.

Parameters ^1^	Patients with Testicular GCT (*n* =229)	Stage I (*n* = 171)	Stage II and III (*n* =57)	*p*-Value ^2^	
Age (years) ^3^	33 (16–61)	33 (16–61)	32 (19–51)	0.758	
The Charlson Comorbidity Index, CCI (value) ^3^	0 (0–1)	0 (0–2)	0 (0–1)	0.417	
Body mass index (kg/m^2^) ^3^	25.4 (17.6–46.4)	25.5 (17.9–37.00)	25.2 (18.5–46.4)	0.938	
Parameters ^1^	Patients with testicular GCT, *n* (%)	Stage I *n* (%)	Stage II and III *n* (%)	OR (95% CI)	*p*-value ^4^
<30 kg/m^2^	168 (88)	125 (88)	43 (84)	1.00 (reference group)	
>30 kg/m^2^	25 (12)	17 (12)	8 (16)	1.368 (0.551–3.395)	0.499
Smoking status					
Never	110 (55)	87 (60)	23 (43)	1.00 (reference group)	
Ever	88 (45)	58 (40)	30 (57)	1.957 (1.035–3.698)	0.039 *
Tumor size					
<4 cm	126 (67)	104 (75)	22 (44)	1.00 (reference group)	
>4 cm	62 (33)	34 (25)	28 (56)	3.893 (1.973–7.681)	<0.001 *
Multifocality					
No	157 (83)	107 (79)	50 (93)	1.00(reference group)	
Yes	32 (17)	28 (21)	4 (7)	0. 306 (0.102–0.919)	0.035 *

^1^ Available data; ^2^ Mann–Whitney U test; ^3^ Median (Min-Max); ^4^ Logistic regression, * *p* < 0.05; Patients were classified as obese if their body mass index (BMI) was more than 30 kg/m^2^, while smokers were defined as individuals who smoked daily for any 60-day interval previous to the start of the trial; GCT-germinative cell tumors; OR-crude odds ratio; CI-confidence interval.

**Table 3:** The difference in preoperative hematological and nutritional parameters, along with inflammatory and immuno-nutritional scores, between testicular GCT patients at lower and higher tumor stages.

Laboratory Parameters	Stage I	Stages II + III	*p*-Value ^1^
Lymphocytes (*n* × 10^9^/L)	1.91 (0.80–4.10)	1.70 (0.40–3.90)	0.401
Platelets (*n* × 10^9^/L)	237.00 (102.00–412.00)	261.00 (169.00–562.00)	0.003 *
Hemoglobin (g/L)	154 (104–181)	147 (83–174)	0.025 *
Albumin (g/L)	48.5 (38–54)	47 (34–56)	0.006 *
HALP	58.84 (16.22–183.88)	45.68 (9.08–103.89)	0.001 *
PNI	57.67 (43.50–72.00)	55.50 (42.00–64.00)	0.040 *

HALP-hemoglobin, albumin, lymphocytes and platelets score (formula: hemoglobin × albumin × lymphocytes/platelets); PNI-prognostic nutritional index (formula: albumin value + (5 × peripheral blood lymphocyte count)); ^1^ Mann–Whitney U test. * *p* < 0.05.

**Table 4:** The discriminating potential of the assessed inflammatory-nutritional scores.

Inflammatory Scores	Cut-Off Points	AUC	*p*-Value	95% CI	Specificity (%)	Sensitivity (%)
HALP	42.56	0.609	0.016 *	0.519–0.699	78.76	44.64
PNI	52.5	0.595	0.043 *	0.501–0.688	91.83	29.09

AUC-area under the curve, CI-confidence interval; HALP-hemoglobin, albumin, lymphocytes and platelets score (formula: hemoglobin × albumin × lymphocytes/platelets); PNI-prognostic nutritional index (formula: albumin value + (5 × peripheral blood lymphocyte count)); * *p* < 0.05.

**Table 5:** The association between different clinicopathological parameters and immuno-nutritional scores.

Parameters	HALP Score			PNI SCORE		
	Low	High	*p*-Value ^1^	Low	High	*p*-Value ^1^
Clinical stage, *n* (%)						
Stage I	30 (20)	118 (80)	<0.0001 *	12 (8)	136 (92)	<0.0001 *
Stage II + III	27 (49)	30 (51)		16 (29)	39 (71)	
Tumor size, *n* (%)						
<4 cm	26 (21)	100 (79)	0.004 *	9 (7)	117 (93)	<0.0001 *
>4 cm	25 (40)	37 (60)		16 (26)	46 (74)	
BMI, *n* (%)						
<30 kg/m^2^	52 (31)	116(69)	0.124	24 (14)	144 (86)	0.759
>30 kg/m^2^	4 (16)	21 (84)		3 (12)	22 (88)	
LVI, *n* (%)						
No	25 (27)	68 (73)	0.509	15 (16)	78 (84)	0.476
Yes	30 (31)	66 (69)		12 (13)	84 (87)	
Multifocality, *n* (%)						
Yes	11 (34)	21 (66)	0.471	20 (13)	137 (87)	0.178
No	44 (28)	113 (72)		7 (22)	25 (78)	

HALP-hemoglobin, albumin, lymphocytes and platelets score (formula: hemoglobin × albumin × lymphocytes/platelets); PNI-prognostic nutritional index (formula: albumin + 0.0005 × lymphocytes); BMI-body mass index, LVI-lymphovascular invasion; High HALP score > 42.56; high PNI score >52.5; ^1^ χ^2^ test; * *p* < 0.05.

**Table 6:** The risk of developing clinically advanced disease in relation to the levels of immuno-nutritional scores.

Parameter	Stage I, *n* (%)	Stage II and III, *n* (%)	OR1 (95% CI)	*p*-Value	OR2 (95% CI)	*p*-Value
High HALP	118 (80)	28 (51)	1.00 (reference group)	<0.001 *	1.00 (reference group)	0.001 *
Low HALP	30 (20)	27 (49)	3.793 (1.954–7.363)		4.161 (1.862–9.269)	
High PNI	136 (92)	39 (71)	1.00 (reference group)	<0.001 *	1.00 (reference group)	
Low PNI	12 (8)	16 (29)	4.650 (2.030–10.650)		5.556 (1. 969–15.677)	0.001 *

High HALP score > 42.56; high PNI score > 52.5; OR1- crude odds ratio; OR2 is adjusted to tumor size (greater than 4 cm), smoking status and multifocality; CI-confidence interval; HALP-hemoglobin, albumin, lymphocytes and platelets score (formula: hemoglobin × albumin × lymphocytes/platelets); PNI-prognostic nutritional index (formula: albumin value + (5 × peripheral blood lymphocyte count)); * *p* < 0.05.

## Data Availability

The data supporting the reported results can be found upon request in the form of datasets available at the Clinic of Urology, University Clinical Centre of Serbia.
